# Local availability of cannabis retail outlets on parents’ cannabis use, acceptability, and perceived addictiveness: A longitudinal study in Washington State

**DOI:** 10.1371/journal.pone.0348182

**Published:** 2026-05-27

**Authors:** Vi T. Le, Jennifer A. Bailey, Marina Epstein, Matthew D. Dunbar, A. Karryn Satchell, Danielle M. Pandika

**Affiliations:** 1 Social Development Research Group, School of Social Work, University of Washington, Seattle, Washington, United States of America; 2 Center for Studies in Demography and Ecology, University of Washington, Seattle, Washington, United States of America; Fundación Universitaria del Área Andina, COLOMBIA

## Abstract

**Objective:**

Parents represent a growing yet understudied population of people who use cannabis in the era of legalization. Studies examining how increasing availability of cannabis retail stores affects parental cannabis use, norms, and perceived harm are needed to guide interventions to support families. This longitudinal study examines whether perceived and objectively measured cannabis outlet availability are associated with parents’ cannabis use frequency, acceptability, and perceived addictiveness in Washington State, where nonmedical cannabis retail stores opened in 2014.

**Method:**

Parents (n = 471) in the Seattle Social Development Project – The Intergenerational Project were surveyed in 2015, 2016, and 2017. Multilevel models were used to estimate within- and between-person associations between (a) perceived cannabis outlet availability (self-reported number of outlets in one’s neighborhood) and (b) objective cannabis outlet availability (number of outlets within a 10-minute drive time from home) with past-year cannabis use frequencies, acceptability of parental cannabis use, and perceived addictiveness.

**Results:**

Local retail outlet availability was higher in later waves. There were significant between-person associations, such that parents residing in neighborhoods with greater cannabis outlet availability (both perceived and objectively measured) over the course of the study had greater cannabis use frequency, higher acceptability of use, and lower perceived addictiveness. When comparing the same parents over time, the association between objectively measured outlet availability and acceptability of use was replicated in the within-person analysis, such that parents had greater acceptability of parental cannabis use in years when they lived in neighborhoods with higher objective outlet availability.

**Conclusions:**

Legal cannabis retail stores are increasingly common within neighborhoods. Prevention programming to support families residing in neighborhoods with greater outlet availability may reduce permissive cannabis norms and use among parents, which can have strong implications for youth substance use prevention and positive youth development.

## Introduction

Current evidence shows that more parents are using cannabis now that it is legal, with prevalence and frequency of use higher among parents residing in legalized states than those without legalization [1–3]. Norms and attitudes surrounding cannabis among parents have also changed due to legalization, with research suggesting increasing acceptability of use and lower perceived harm [1,2,4]. The implications of these trends are concerning due to decades of research highlighting the risk parental cannabis use poses for families and the important role parents play in modeling behaviors and norms for youth’s substance use, even in the absence of parental use [5–8]. Prior research suggests that laws permitting cannabis sales are more strongly associated with adult cannabis use than the simple enactment of legalization [9,10], yet ways in which post-legalization increases in the availability of cannabis stores relate to parents’ cannabis use, norms, and perceived harm are understudied.

Existing studies suggest that greater cannabis outlet availability in one’s residential neighborhood is associated with higher prevalence and frequency of cannabis use among adults in general; however, longitudinal studies examining parental cannabis use behaviors and norms are rare [11–15]. Availability theory purports that greater physical availability of a product, such as a higher number of retail outlets, leads to greater sales and consumption of a product [16]. Greater availability of cannabis retail stores may affect cannabis consumption levels by reducing travel distance/time to obtain products and by offering consumers a greater diversity of products at lower prices [17,18]. Additionally, social cognitive theory suggest that presence of retail outlets may alter other environmental factors, including advertising, access, and the promotion of more permissive norms that can alter cannabis use behaviors [19]. Living near more medical cannabis outlets has been associated with more positive expectancies of cannabis use [e.g., helps with relaxation, more fun; [20], which are then associated with prevalence and frequency of use among adults [21–23]. However, much of the current research examining the effects of cannabis retail outlets has been cross-sectional [15]. Longitudinal studies with repeated assessments of neighborhood retail environments and cannabis use behaviors could allow testing of whether changes in local retail outlet availability are associated with changes in individual behavior over time and whether parents exposed to more cannabis outlets tend to use more cannabis and have more permissive norms.

Neighborhood effects research shows that objective and subjective (or perceived) assessments of the neighborhood are both important factors that influence health behaviors [24]. Yet, research examining local cannabis outlet availability to date has mainly focused on objective measures of availability, such as the density of cannabis retail outlets surrounding one’s residence [25]. While objective measures can capture the number of outlets within a defined area, individuals’ behavior and attitudes may not be affected by outlets that they are not exposed to or don’t interact with or, conversely, individuals who use cannabis may be more aware of outlets in their neighborhood. Studies that ask participants about the presence of cannabis outlets in their neighborhood may provide an accurate reflection of an individual’s exposure to cannabis stores as they navigate through daily life [24,26]. Evidence suggests that perceived and objective assessments of neighborhood characteristics are often positively – although not highly – correlated with each other, with participants reporting different neighborhood sizes and features, compared to those obtained from GIS-based assessments [24,27,28]. Studies that examine both perceived and objective measures of cannabis outlet availability may provide insight into how different measures of outlet availability relate to cannabis use behaviors and norms.

Using data from a longitudinal cohort of parents, the goal of the current study is to examine associations between repeated perceived and objective measures of neighborhood cannabis retail outlet density with parents’ self-reported use frequency, acceptability, and perceived addictiveness of cannabis use. Analyses examined the influence of cannabis retail exposure on both individual changes in behavior and changes between different groups of parents. We hypothesized that perceived and objective measures of cannabis retail outlet availability would be associated with parents’ self-reported use frequency, acceptability, and perceived addictiveness of cannabis use at both the within-and between-person level. At the between-person level, parents in neighborhoods with higher outlet availability would have greater cannabis use and more permissive norms. At the within-person level, increases in outlet availability would be associated with greater cannabis use and more permissive norms within the same individual over time.

## Materials and methods

### Study design, participants, and setting

Data were drawn from the Seattle Social Development Project – The Intergenerational Project (SSDP-TIP), an accelerated longitudinal cohort of Washington families since 2002. SSDP-TIP participants were recruited from the Seattle Social Development Project, which enrolled elementary students from 18 public schools in Seattle, Washington in 1985. Recruited participants included SSDP participants who became parents (“SSDP parents”), their oldest biological child with whom they had regular contact, and another caregiver (usually the spouse, but sometimes other relatives or an unrelated adult) who shared responsibility for raising the child [1,29]. Recruitment for SSDP-TIP began February 1, 2001 and ended October 1, 2025. Ten waves of interviews from 426 families have been collected. Parents provided informed consent for their own participation and permission for their minor child’s participation at each survey wave. Study procedures are approved by the University of Washington Human Subjects Institutional Review Board.

The current study used 3 waves of SSDP-TIP surveys collected in 2015, 2016, and 2017, which occurred after the state’s legalization of adult nonmedical cannabis use in 2012 and the opening of cannabis retail outlets in 2014. Roughly 91% (n = 389) of SSDP families participated in 2015–2017. To understand parental cannabis use and norms, the current study was restricted to SSDP parents and second caregivers who identified as the other biological parent of the child or the spouse of the SSDP parent (83% of all second caregivers). For these waves, all surveys for parents and caregivers were conducted on the web and electronic, informed consent was obtained. Inclusion was further restricted to 315 families who primarily resided in Washington. Two families were excluded due to the use of a post office box as their mailing address. A total of 471 parents (n = 293 SSDP parents, n = 178 second caregivers) were eligible for the current study. Roughly 91% of second caregivers in the analytic sample identified as the other biological parent, while 9% identified as a stepparent.

### Measures

#### Perceived Cannabis retail availability.

At each wave, parents were asked to “think about the neighborhood where [they] live” and report the number of marijuana stores in their neighborhood (0 = “none,” 1 = “one”; 2 = “two or three”; 3 = “four or five”; 4 = “six or more”).

#### Objective Cannabis retail outlet density.

Primary residential addresses of parents and caregivers were obtained during the 2015 and 2016 assessments. To determine objective cannabis outlet density, residential addresses were geocoded using ArcGIS, with Esri Business Analyst reference data. Addresses were matched to either rooftop or street-address interpolated reference data using a minimum match score ≥ 95. Spatial coordinates for each participant were used to determine measures of retail cannabis outlets at fixed street network buffers by incorporating spatial data obtained from the Washington State Liquor and Cannabis Board on cannabis retailers operating in 2015 and 2016.

Objective density was characterized as the number of licensed cannabis retailers within a 10-minute driving buffer around each participant’s residence, which considers street geography relevant in travel behaviors. Similar metrics are used in other studies examining cannabis retail outlet density [13,20,30,31]. The 10-minute buffer chosen was based on national data showing that most households drive an average of 3.8 miles or about 10 minutes to obtain consumer goods [32,33].

#### Parent Cannabis use frequency.

Parents and second caregivers self-reported their cannabis use frequency in the past year at each wave. Frequency of use was categorized as 0 = “Never”, 1 = “Monthly or less,” 2 = “Weekly,” or 3 = “Daily or more”.

#### Acceptability of parental Cannabis use.

At each wave, the measure of parents’ acceptability of cannabis use combined two items which asked participants to rate whether they thought it was “okay for parents to use marijuana occasionally” and “okay for parents to use marijuana regularly.” Response options used a Likert-type 4-point response format: 0 = “NO!,” 1 = “no,” 2 = “yes,” 3 = “YES!.” Responses were averaged across items within waves, such that higher scores indicated greater acceptability of parental cannabis use (Cronbach’s alphas 0.87–0.89 across waves).

#### Perceived Cannabis addictiveness.

At each wave, parents and caregivers were asked: “Do you think that marijuana is addictive?”. Response options included 0 = “NO!,” 1 = “no,” 2 = “yes,” 3 = “YES!.” Higher scores indicate that participants thought cannabis was more addictive.

#### Covariates.

Parents and second caregivers self-reported their age, sex (male, female), educational attainment at the time of study entry (no high school diploma, high school graduate, 4-year college graduate), and race (Asian American or Pacific Islander; Black or African American; White; or other races including Native American or multiracial). Cannabis retail outlets are often located within neighborhoods with more racial and ethnic minority populations and higher economic disadvantage, with some evidence linking neighborhood disadvantage with increased cannabis use and acceptability [11,34,35]. As such, analyses controlled for neighborhood disadvantage using the Area Deprivation Index (ADI). The ADI is a validated, neighborhood-level composite index aggregating 17 measures of education, employment, poverty, and housing quality obtained from the 2010–2015 American Community Survey at the census block group level [36–38]. Raw composite scores are converted to deciles (1–10), where higher numbers represent higher levels of neighborhood disadvantage.

### Statistical analysis

To examine associations between cannabis outlet availability and cannabis outcomes, multilevel modeling was used to account for clustering of observations within individuals and within families. The effects of perceived and objective measures of outlet availability were examined separately. Analyses examining objective outlet availability were restricted to data from two waves (2015 and 2016). To disaggregate within- and between-person effects, models included cannabis retail outlet density measures as both time-varying and time-fixed predictors. At level 1, cannabis outlet density was group-mean centered to represent the difference from an individual’s age-specific report of cannabis outlet availability and their average outlet availability across the study period. The regression coefficient estimates how cannabis use and acceptability are related to deviation in cannabis outlet availability around the individual’s mean level of outlet availability across all years (within-person effects). At level 2, cannabis outlet availability was grand-mean centered and represents the mean level of outlet availability across all available years of data for a given person. The regression coefficient represents whether parents exposed to higher levels of outlet availability, compared to the sample’s average, had higher levels of cannabis use frequency, higher acceptability of parental use, and lower levels of perceived harm (between-person effects). Models were estimated using Mplus version 8.4 with Maximum Likelihood estimation with robust standard errors for acceptability of parental cannabis use (continuous outcome) and with logit links for cannabis use frequency and perceived harm (categorical outcomes). Repeated measures were arrayed by parent age (centered at 41 years), and linear changes in cannabis use, acceptability, and perceived harm were modeled across time. Models accounted for clustering of parents within the same family using the *TYPE = COMPLEX* option. Analyses adjusted for time-varying age and time-fixed controls, such as sex, race and ethnicity, educational attainment, and neighborhood disadvantage. Sensitivity analyses explored alternative metrics of objective cannabis retail availability by using the number of outlets within 5- and 15-minute driving buffers. Data for the current analyses were accessed from March 4, 2024 to September 21, 2025. Participants’ identifying information (e.g., birth dates, residential addresses) was stored separately from the analytic data and was only accessible by the Principal Investigator of SSDP-TIP (second author).

## Results

Table 1 shows characteristics of the study sample. Across all observations, average age ranged from 36 to 46 years, with roughly 12.3% of observations between 36–39 years old, 70.8% between 40–42, and 16.9% between 43–46 years old. Roughly 6% of parents and caregivers in the sample identified as Hispanic (not mutually exclusive with other racial categories). Across all observations, most parents (76.1%) did not use cannabis in the past year, while 9.9% reported monthly use or less, 7.2% reported weekly use, and 6.8% reported daily use. On average, there was low acceptability of parental use (mean = 1.08; SD 0.92; range: 0–3), with most parents (78%) endorsing that “cannabis was addictive” (mean = 2.22; SD: 0.91, range: 0–3). There was substantial within-person correlation in cannabis use frequency (intraclass correlation = 0.79), acceptability of parental use (intraclass correlation = 0.73), and perceived harm (intraclass correlation = 0.69) across all observations.

**Table 1:** Demographics of parents in SSDP-TIP, N = 471.

Variables	N (%)
Age, mean (SD)	40.4 (1.8)
Sex	
Female	272 (57.7)
Male	199 (42.3)
Race and ethnicity	
Asian or Pacific Islander	93 (19.9)
Black or African American	82 (17.6)
Native or Multiracial	74 (15.8)
White	218 (46.7)
Educational attainment	
No high school diploma	75 (16.0)
High school graduate	255 (54.5)
4-year college graduate	138 (29.5)
Area Deprivation Index, mean (SD)	3.4 (2.4)

On average, local cannabis outlet availability was higher in later waves. Roughly 43.0% of parents reported having at least one cannabis outlet in their neighborhood in 2015, compared to 52.8% in 2017. Moreover, the average number of outlets within a 10-minute drive from one’s residence increased from 2.7 outlets (SD = 3.0) in 2015 to 4.6 outlets (SD = 4.5) in 2016. Across all waves, around 57.7% and 65.2% of participants experienced a change in their perceived and objective cannabis retail outlet density, respectively. A weak-to-moderate correlation was noted between perceived number of outlets and objectively measured outlet availability within a 10-minute drive time (correlation = 0.28). Nearly half of participants (49.2%) self-reported having at least one cannabis retail outlet in their neighborhood over the study period. In contrast, geocoded data show that 77.5% of participants had at least one cannabis retail outlet located within a 10-minute drive of their residence.

Multilevel regression models showed significant between-person associations, indicating that, on average, parents who perceived higher cannabis outlet availability over the course of the study had greater cannabis use frequency, higher acceptability, and lower perceptions of addictiveness Table 2). When comparing within the same individuals over time, perceived cannabis availability was not significantly associated with any cannabis outcomes. That is, parents were not more likely to use cannabis or expressed more permissive cannabis norms in years where they perceived a higher number of outlets in their neighborhood.

**Table 2:** Multilevel regression models for the adjusted associations between perceived cannabis outlet availability and cannabis use outcomes.

	Past-year cannabis use frequency		Acceptability of cannabis use		Perceived cannabis as addictive	
	β (SE)	p-value	β (SE)	p-value	β (SE)	p-value
**Within-person**						
Age	−0.02 (0.10)	0.820	0.02 (0.06)	0.692	−0.05 (0.06)	0.384
Perceived outlet availability	0.07 (0.06)	0.274	0.04 (0.03)	0.193	0.01 (0.04)	0.820
**Between-person**						
Perceived outlet availability	**0.24 (0.05)**	**<0.001**	**0.31 (0.05)**	**<0.001**	**−0.24 (0.05)**	**<0.001**
Female (ref. male)	**−0.20 (0.09)**	**0.036**	**−0.22 (0.08)**	**0.011**	**0.25 (0.09)**	**0.006**
Race (ref. White)						
Asian or Pacific Islander	**−0.39 (0.17)**	**0.019**	**−0.56 (0.12)**	**<0.001**	**0.73 (0.15)**	**<0.001**
Black or African American	−0.03 (0.15)	0.815	−0.29 (0.15)	0.052	**0.29 (0.15)**	**0.049**
Native or Multiracial	0.25 (0.16)	0.117	0.08 (0.16)	0.636	−0.02 (0.17)	0.918
Educational attainment	**−0.13 (0.06)**	**0.021**	−0.05 (0.06)	0.377	0.04 (0.09)	0.658
Area Deprivation Index	0.03 (0.06)	0.600	0.00 (0.06)	0.976	0.03 (0.06)	0.591

Note: Estimates are standardized. Bolded estimates are significant at p < 0.05.

Using a 10-minute drive time buffer, the effects of outlet availability differed by cannabis outcomes (Table 3). The between-person association showed that, on average, parents who resided in neighborhoods with greater outlet availability reported significantly higher acceptability (β = 0.16 (0.06); p-value = 0.004) and lower perceived addictiveness (β = −0.14 (0.06); p-value = 0.016) over the course of the study. There were no significant within- or between-person associations between objective cannabis outlet availability and use frequency. There were both within- and between-person associations with acceptability of parental use. Specifically, when comparing the same individuals over time, parents displayed higher acceptability of parental cannabis use in years when they lived in neighborhoods with greater outlet availability (within-person effect: β = 0.09 (0.04); p-value = 0.018).

**Table 3:** Multilevel regression models for adjusted associations between objective cannabis outlet availability within 10-minute drive time and cannabis use outcomes.

	Past-year cannabis use frequency		Acceptability of cannabis use		Perceived cannabis as addictive	
	β (SE)	p-value	β (SE)	p-value	β (SE)	p-value
**Within-person**						
Age	−0.02 (0.14)	0.909	−0.06 (0.07)	0.390	**0.18 (0.08)**	**0.035**
Objective outlet availability	0.04 (0.06)	0.536	**0.09 (0.04)**	**0.018**	−0.04 (0.05)	0.433
**Between-person**						
Objective outlet availability	0.08 (0.06)	0.220	**0.16 (0.06)**	**0.004**	**−0.14 (0.06)**	**0.016**
Female (ref. male)	**−0.25 (0.10)**	**0.013**	**−0.26 (0.09)**	**0.005**	**0.36 (0.10)**	**0.007**
Race (ref. White)						
Asian or Pacific Islander	**−0.48 (0.19)**	**0.011**	**−0.63 (0.13)**	**<0.001**	**0.83 (0.16)**	**<0.001**
Black or African American	0.09 (0.16)	0.588	−0.21 (0.16)	0.195	0.25 (0.17)	0.127
Native or Multiracial	0.31 (0.17)	0.063	0.23 (0.17)	0.173	−0.03 (0.18)	0.850
Educational attainment	**−0.13 (0.06)**	**0.045**	−0.02 (0.06)	0.699	−0.03 (0.10)	0.724
Area Deprivation Index	0.04 (0.07)	0.541	0.04 (0.07)	0.607	−0.01 (0.07)	0.929

Note: Estimates are standardized. Bolded estimates are significant at p < 0.05.

Sensitivity analyses incorporating the number of cannabis retailers in smaller (5-minute) and larger (15-minute) driving buffers showed consistency when compared to findings using the primary measure of 10-minute driving buffer (Table 4). No significant within-person changes in cannabis use frequency or perceived addictiveness were found at any of the drive-time buffers. Between-person analyses suggested stronger associations with larger spatial buffers compared to the more proximal buffers. Specifically, results from between-person analyses showed that the number of stores within a 15-minute drive was associated with cannabis use frequency; however, no statistically significant associations were observed using smaller driving buffers.

**Table 4:** Sensitivity analyses examining adjusted associations between varying metrics of objective cannabis outlet availability and cannabis outcomes.

	Past-year cannabis use frequency		Acceptability of cannabis use		Perceived cannabis as addictive	
	β (SE)	p-value	β (SE)	p-value	β (SE)	p-value
**Within-person**						
Stores within 5-minute	0.09 (0.07)	0.159	**0.08 (0.03)**	**0.018**	−0.02 (0.05)	0.725
Stores within 10-minute	0.04 (0.06)	0.536	**0.09 (0.04)**	**0.018**	−0.04 (0.05)	0.433
Stores within 15-minute	0.01 (0.07)	0.936	**0.09 (0.04)**	**0.027**	−0.02 (0.05)	0.731
**Between-person**						
Stores within 5-minute	0.02 (0.06)	0.717	**0.11 (0.05)**	**0.029**	**−0.15 (0.05)**	**0.002**
Stores within 10-minute	0.08 (0.06)	0.220	**0.16 (0.06)**	**0.004**	**−0.14 (0.06)**	**0.016**
Stores within 15-minute	**0.15 (0.06)**	**0.010**	**0.20 (0.06)**	**<0.001**	**−0.17 (0.06)**	**0.003**

Note: Estimates are standardized. Bolded estimates are significant at p < 0.05.

## Discussion

An increasing number of residents in the United States are living near legal cannabis retail stores [39], highlighting the need to examine the effects of increasing cannabis outlet availability on patterns of cannabis use and norms. Of particular concern, cannabis use is becoming increasingly prevalent among parents [1–3], who have historically served as avenues for intervention to reduce youth cannabis use and promote positive youth development. The current study aimed to examine within- and between-person associations between perceived and objective measures of neighborhood cannabis retail outlet density with parents’ self-reported use frequency, acceptability, and perceived addictiveness of cannabis use. This study found that parents residing in neighborhoods with more cannabis retail outlets on average tended to have higher acceptability of cannabis use and were less likely to view cannabis as addictive (between-person effect). Within-person analyses comparing the same individuals over time showed that parents reported greater cannabis use acceptability, but not use frequency or perceived addictiveness, in years where they had higher objective retail outlet availability.

Findings from this study suggest that perceived and objectively measured cannabis outlet environments both have implications on parenting drug norms, attitudes, and use. For example, significant between-person associations were found for both perceived and objective cannabis retail outlet measurements with regards to cannabis acceptability of use and perceived addictiveness. These finding are consistent with and extend previous studies linking cannabis outlet availability with lower perceived harms and higher perceived acceptability of cannabis among youths and young adults [11,40]. Study results were a bit more nuanced regarding cannabis use frequency. Perceived outlet availability was positively associated with use frequency, suggesting that individuals who use cannabis may frequent retails outlets and therefore simply be more aware of outlets in their neighborhood. Notably, the current study did not find between-person associations between neighborhood outlet availability within 10-minute drive buffer (roughly 4 miles) and cannabis use. This is in contrast to prior cross-sectional studies which found associations between cannabis outlet availability and use at smaller buffers, such as less than 1 mile [11,12], and larger buffers at 4 and 5 miles from participant’s homes [13,14,20,41]. In the current study, sensitivity analyses incorporating various driving-distance buffers showed a systematic increase in parameter estimates and corresponding decrease in p-values observed as drive time buffers increased from 5 to 15 minutes. This suggests that there may be some density/distance threshold or u-shaped relationship between drive time/outlet availability and cannabis use among parents. These possible relationships should be tested and may have regulatory implications useful for determining where cannabis outlets should be located in order to mitigate harms. In addition, norms surrounding cannabis are strongly linked to actual use, and changes in use may require longer follow-up to emerge.

Results shows that most of the associations linking cannabis outlet availability to cannabis outcomes were found at the between-person level, with few within-person associations found. Specifically, when comparing the same parents over time, there was a significant – albeit weaker – effect of objectively measured outlet availability on acceptability of use, which may suggest potential independent effects of the cannabis retail environment on acceptability of use. However, no other within-person effects were found. While many parents experienced changes in local outlet availability over the course of the study, studies incorporating more repeated measures of outlet availability over a longer follow-up period may have greater power to detect within-person effects. Given that family structure may shape opportunities, norms, and constraints around use, future research could examine whether retail environment influences vary across family composition and the downstream implications for youth outcomes. Nonetheless, findings from this study suggest that parents residing in neighborhoods with more outlets had more permissive attitudes and frequent use surrounding cannabis. Thus, reducing the concentration of cannabis retailers and placing restrictions on environmental cues (e.g., storefront advertising) may mitigate the market’s influence on parental cannabis use acceptability and related harms [42].

Current findings raise concerns about the implications of the cannabis retail environment on youth, as mediated through parental use and norms. Numerous studies conducted before legalization have shown associations between parent cannabis use and acceptability with offspring substance use, including cannabis [7,43]. Parents can play a key role in communicating permissive rules, beliefs, and norms surrounding cannabis use to their children, which can strongly influence youths’ cannabis norms and use behaviors [44,45], even in the absence of parental use [8]. Qualitative surveys of parents in Washington show that many parents are using cannabis now that it is legal, with concerns about the implications of cannabis legalization for their children [46]. As such, prevention programs that equip parents with strategies on how to communicate to their children about cannabis and counter prevailing social norms in the context of legalization will be important as cannabis becomes more normalized [46]. Since youth cannabis use is associated with adverse life outcomes, including later dependence, lower academic attainment and income, and justice system involvement [47], continued research on the implications of cannabis legalization and increased availability on parents and their children is needed to guide cannabis policy.

Several limitations should be considered when interpreting these findings. The sample was drawn from Washington State, and results may not be generalizable to areas with different policies for retail cannabis sales (e.g., more liberal marketing and advertising, fewer regulations on hours or location of stores). Follow-up was restricted to 3 years after the opening of the cannabis retail market in Washington, with objective measures available to 2 years of follow-up; thus results may not capture the full impact of the retail cannabis market [48]. However, growth in retail store openings has slowed to <1% per month in Washington since 2017, with the greatest increase in number of stores occurring in the first few months of market opening in 2014 [49]. Information on the illegal drug market, such as unlicensed cannabis sources (e.g., dealers, home grows), was not available and may confound any effects of licensed retail outlet availability; however, around 75% of Washington adults who purchase cannabis typically buy it from licensed retail stores [11,50]. Frequency of cannabis use was analyzed as a categorical variable, consistent with previous work [51,52]. Future studies would benefit from incorporating more granular measures of use (e.g., number of days used, quantity, potency, or mode of use). Residential self-selection is possible, such that parents who use cannabis and have more permissive cannabis norms may choose to live closer to cannabis retail outlets. Testing of within-person associations could have mitigated potential biases related to neighborhood selection or other unmeasured factors [53–55]. Lastly, parents may be exposed to retail outlets in places outside their residential neighborhoods; thus, future work should consider other activity spaces where cannabis retailers may be available, such as around work and recreational settings. Despite these limitations, our study adds several contributions: (a) using longitudinal data to examine changes in outlet density, use, and norms over time; (b) including both perceived and objective measures of neighborhood cannabis retail environments; and (c) incorporating sensitivity analyses with various driving-distance buffers.

## Conclusions

To inform state and local regulatory policies, evaluating the influence of characteristics of retail cannabis markets, such as outlet availability, will continue to be important as cannabis retail markets expand. Findings from this longitudinal study of parents in Washington State suggest that at the between-person level, parents residing in neighborhoods with greater cannabis outlet availability reported more permissive cannabis norms and greater use. At the within-person level, parents displayed higher acceptability of cannabis use when they lived in neighborhoods with greater outlet availability. Policies restricting density may be an effective leverage point in reducing parent pro-use norms and use, which has important implications in prevention of youth cannabis use.
